# Cultures that collide: an ethnographic study of the introduction of a palliative care consultation team on acute wards

**DOI:** 10.1186/s12904-021-00877-1

**Published:** 2021-11-21

**Authors:** Maria Friedrichsen, Yvonne Hajradinovic, Maria Jakobsson, Kerstin Brachfeld, Anna Milberg

**Affiliations:** 1 Palliative Education and Research Centre in Region Östergötland, Vrinnevi Hospital, 601 82 Norrköping, Sweden; 2 Department of Nursing Science, Sophiahemmet University, Stockholm, Sweden; 3 Department of Palliative Medicine, Vrinnevi Hospital, Norrköping, Sweden; 4 Department of Health, Medicine and Caring Sciences, Division of Prevention, Rehabilitation and Community Medicine, Linköping University, Linköping, Sweden

**Keywords:** Palliative care consultation team, Organisational culture, End-of-life care, Acute wards, Hospital

## Abstract

**Background:**

Acute care and palliative care (PC) are described as different incompatible organisational care cultures. Few studies have observed the actual meeting between these two cultures. In this paper we report part of ethnographic results from an intervention study where a palliative care consultation team (PCCT) used an integrative bedside education approach, trying to embed PC principles and interventions into daily practice in acute wards.

**Purpose:**

To study the meeting and interaction of two different care cultures, palliative care and curative acute wards, when a PCCT introduces consulting services to acute wards regarding end-of-life palliative care, focusing on the differences between the cultures.

**Methods:**

An ethnographic study design was used, including observations, interviews and diary entries. A PCCT visited acute care wards during 1 year. The analysis was inspired by Spradleys ethnography.

**Results:**

Three themes were found: 1) Anticipations meets reality; 2) Valuation of time and prioritising; and 3) The content and creation of palliative care.

**Conclusion:**

There are many differences in values, and the way PC are provided in the acute care wards compared to what a PCCT expects. The didactic challenges are many and the PC require effort.

## Introduction

In health care, different care cultures are described. One is the organisational culture that is defined as: “the values, behaviours, goals, traditions, attitudes, practices and beliefs shared across an entire organisation [1] which are deeply ingrained in the everyday life of the organization and its members [2]”. Another culture is the workplace culture: “a specific type of subculture involving an identifiable grouping within an organisation. In healthcare, such a ‘workplace’ may be a unit, ward, department, or a professional group, e.g., medicine or nursing” [1].

Sometimes, acute care and palliative care (PC) are described as different incompatible cultures, with contrasts and contradictions [3]. The biomedical, acute culture involves “adding or continuing all life-sustaining therapies” [4]. On acute wards [5], the logic of care is motivated by limited resources and the demanding medical unit create a context of constant activity. Hierarchies and priorities influence roles, routines and interactions, as well as how primary care team members approach different patient groups. The vast workload results in priorities, and assumes the importance of curative care over PC. Team members prioritise life-prolonging activities, and are less attentive to care of dying patients. Important outcomes for acute wards are mortality rates, failure to rescue, readmission rates and adverse events/medication errors [1].

In contrast, PC has its origins in philosophy and is an interdisciplinary approach with importance given to all members of the multi-professional team. The patient and his/her family, rather than the patient alone, are the essential unit of care. Care should be holistic where physical, psychological, social, spiritual, and existential needs are in focus [6]. It is of value to have conversations about end of life, as death is not a failure. Important outcomes in PC are the patient-reported outcome measures regarding physical symptoms, emotional, psychological and spiritual needs, provision of information, and support of quality of life [7, 8].

A question exists whether there any benefits to introducing palliative care consultation team (PCCT) on acute wards. When introducing a PCCT on acute wards, patient satisfaction has improved [9] as well as perceptions of well-being [9, 10]. Different studies have shown improvement in symptom distress [11–16] as well as no difference/improvements in symptom scores [17]. They also assists in that fewer patients are less admitted to, or die in intensive care units, as patients received less invasive procedures, and costs was thereby reduced [18, 19]. The PCCT also influences the reduction of potentially inappropriate medications [20]. There are also qualitative data regarding implementation of a PCCT in acute wards. Studies have focused on primary team members’ knowledge of and attitudes towards PC and a PCCT [21], including expectations [22, 23], collaboration, and partnership [24, 25].

However, patients die despite health care organisation or culture. In Sweden, 39% of all patients that die an expected death, decease in hospital [26]. Therefore, it is important that all health care personnel have basic skills in PC to care for these patients. Previously, unmet PC needs among patients dying in hospital were identified [25, 27–29]. It is also known that team members in settings other than specialised PC may not prioritise, do not recognise, or are uninterested in PC competencies [25, 30] and have deeply rooted beliefs in their self-sufficiency [21]. It is therefore a delicate didactic question whether it is possible for PC competencies to influence the acute care culture about PC in the end of life.

The current study reports parts of ethnographic results from a larger project [31], where the focus has been to implement a PCCT on internal medicine and surgical wards where general internal medicine patients (cancer, lung diseases, kidney diseases) and general surgical patients (cancer, GI diseases, urological) in all phases of their disease were cared for. A regional multidisciplinary team developed the intervention, with the purpose of enabling the acute healthcare team members to make positive changes in their clinical practise. The quantitative study showed significant changes regarding the primary team members’ perception of quality of communication with patients and their family members and in good quality in end-of-life care. No significant change was seen for adequate symptom relief [31]. Although there is evidence about the benefits and effectiveness of a PCCT, this study will focus on the process when participants in these two cultures meet on acute wards. The aim was to study the meeting and interaction of two different organisational care cultures, PC and curative and acute wards, when a PCCT introduces consulting services to acute wards regarding end-of-life PC, focusing on their differences.

## Methods

### Design

Ethnography is the study of social interactions, behaviours, and perceptions that occur within teams or organisations with origins in anthropology. We chose ethnography as it has an advantage, that it gives the researcher direct access to the culture and practices of a group and can generate rich understanding of the social action that occurs by observing the language, behaviours and values of the participants [32]. The central aim of ethnography is to provide holistic insights into groups of humans’ views and actions, as well as the nature (sights, sounds) of the location they inhabit, through the collection of detailed observations and interviews. In this study, the cultures are a PCCT and team members at acute hospital wards. In this study, Spradleys way of seeing ethnography was used [33], sometimes called ethno semantics, which mainly is based on the idea that language is the primary means that relates meaning in a culture.

### The intervention

In one county in Sweden, the governing politicians requested that high-quality PC should progressively become available to the wider intended group, regardless of where the care is given [34], even in acute wards in hospitals. Therefore, an intervention was developed by a regional multidisciplinary team, designed with the purpose of enabling the primary healthcare team members to make positive changes in their clinical practise and the PC they provided in acute wards. An integrative bedside education approach was used, where physicians and nurses specialised in PC, and experienced in PC, tried to embed PC principles and interventions into daily practice. This intervention was inspired by the pedagogical ideas of Dewey [35, 36], where experience and knowledge are intertwined and developed together. Knowledge creates in social processes together with others and changes without the appearance of chaos. When integrating new knowledge and comparing it with reality and its consequences, people reconstruct their picture of reality. Participants are introduced to the problem, discuss it, argue, negotiate and agree on a solution. Another important aspect in this pedagogical thinking is evaluation.

Specific aims were to identify and emphasise and relieve physical, psychological, social, spiritual, and existential needs in dying patients and their family members on the local wards [6] by educating primary team members in these areas. The PCCT were available for 1–8 daytime hours/week for 1 year (m = 6.5 h/week), visiting the wards and taking part in reports, rounds, and communications. They also identified patients at risk for poor outcomes, who may benefit from a PC consultation. The PCCT supported primary healthcare team members for example when communicating with patients. The primary team could also ask for specific education for all team members in the wards. Education in PC philosophy, pain management and mouth care were provided (total 48 h). The intervention continued through 1 year. To study this intervention a quantitative study was performed with pre-post comparisons to evaluate the primary health care team members’ perceptions about PC on their own ward as well as an ethnographic study to be able to observe the actual meeting, the pedagogical challenges and solutions. This paper reports data from the ethnographic part of the study. For information regarding the quantitative part, of the project see Friedrichsen et al. [31].

### Data collection

The study took place in a local hospital in the south of Sweden with 2400 employees in 2010–2012. Three different wards, one internal medicine ward and two surgical wards were field sites, as these wards should start to involve a PCCT in the daily care. Twenty- eight patients were cared for on each in-patient wards.

The data collection was based on participatory observations, interviews and diary entries written by the PCCT during 1 year (Table 1). The first author, with long experience in different qualitative methods (female associate professor in palliative nursing), collected the data. The observations focused on almost all situations where the PCCT were involved: in the daily routines of the primary team members, actions and roles during rounds, staff meetings, and evaluations and interdisciplinary conferences, but not when working with patients or family members. All field notes were written down in a notebook concerned what was said, how and where. An overt approach was used, where the researcher aimed to integrate into the setting where the purpose of the researcher role is acknowledged. The researcher observes and describes from the outside, i.e. an etic approach. Depending on the situation on the ward, interviews were prepared and audio-recorded, or spontaneous interviews were recorded in the notebook. Questions were posed regarding a specific situation and participant’s experience. Prepared interviews focused on evaluation of the PCCT intervention from the different cultures, where questions were posed regarding the participants positive and negative experiences. The interviews lasted between 3 min to one and a half hours. A total sample of 136 team members participated actively or passively (Table 2).

**Table 1:** Data collection during 2 years

Data collection	H/n
Observations	143
Interviews	35
Notice from diaries	57

**Table 2:** Socio-demographic data of all participants

	Total sample ***n*** = 136
**Gender**	
Males (%)	14 (10)
Females (%)	122 (90)
**Age in years (m)**	41
**Years of experience working in health care**	17.0
**Occupation**	
Physicians (%)	21 (15)
Nurses (%)	73 (54)
Assistant nurses (%)	39 (29)
Others (%)	3 (2)

### Data analysis

In this study, Spradleys methodology [33] inspired the analysis, which mainly (but not only) is based on the idea that language is the primary means that relates meaning in a culture. 1. In the domain analysis, domains (broad categories) were identified by reading through the data material. This included coding words, quotes, situations and reflections [33]. Similar data constituted one domain. Further, this analysis identified relationships between the domains. 2. In the taxonomic analysis, we decided to analyse all domains in depth to get a better understanding of the meaning in the domains. After that, we made a system of classifying and organising the internal organisation of each domain, illustrating the sub-categories of each one. 3. In the componential analysis, the similarities and differences of terms in each domain were compared regarding its relevant features. 4. In the theme analysis, cultural themes are discovered, that is an idea that is specific to a certain *culture*. The established domains were condensed to cultural themes. The analysis was not linear, but moved back and forth between the steps, and during the whole analysis structural and contrasting questions were asked. All data material was reread; themes, domains, situations and quotes deemed, relevant for the analysis [37] was identified, and this process continued until no further information emerged. The first author was the main analyser and the other authors questioned the analysis in order to triangulate the data from different researcher perspectives. Finally, the results were presented to and discussed with the primary nurses and assistant nurses as a member checking of the observations, and the interpretations of them, at a workplace meeting (no physicians participated). This was used as a form of triangulation. Participants recognised the results and further discussions focused on practicalities regarding PC.

### Ethical considerations

The study was conducted according to the Declaration of Helsinki. The regional ethical review board assessed this project as a quality improvement project and ethical approval was therefore given by the head of the clinics at a local hospital (ZZ hospital, internal medicine clinic and, surgical clinic 2010–2011). Patients were part of the intervention, but did not participate in the study, as the study focused on the PCCT and primary team members. All team members were informed verbally and in writing of the purpose of the study, their voluntariness and their right to refrain without explanation, and assured of confidentiality in analysis and publications. The wards and the hospital were anonymised. No one declined to participate.

## Results

The three thematic areas that arose from our analysis were: Anticipations meets reality; Valuation of time and prioritising; and; The content and creation of palliative care (Table 3).

**Table 3:** Overview of the themes

- Anticipations meets reality	
- Valuation of time and prioritising	
- The content and creation of palliative care	

### Anticipations meets reality

Before the start of the intervention, the PCCT anticipated that they would have to defend the project, as the primary team members may be a little hesitant and suspicious. They were surprised about the initial reception at the wards.

*Today we started with the group of physicians… who were all there! Even the head nurses and the head of the clinic were there! We presented our project. We got a very good response and a good discussion with all the physicians. We feel strengthened, welcome and look forward to start the work tomorrow!* Diary, PCCT, nurse X (F= female) and consultant B (F).

On the acute wards, primary team members had different expectations about the PCCT. There was a willingness to learn more about PC, especially symptom management while some team members could not see the point of a PCCT on the acute wards, as they already had the skills needed to take care of dying patients. In the primary teams, some expected that only nurses and assistant nurses should handle PC, or else specialist PC units, and did not see it as their own responsibility, since they had other important work, i.e. focus on the patients that they could help and cure.

*Primary consultant SW 1 (F): This patient should be transferred to the specialised PC unit (looks at the PCCT)!*

*PCCT, nurse X (F): But that is not our task here.*

*Primary consultant SW 1(F): Then, why are you here?*

*PCCT nurse X (F): To implement PC thinking here, in this ward.*

*Primary consultant SW 1(F): We are already very good at PC (sighs, disappointed).* Observation from a round.

Most primary nurses and assistant nurses showed an understanding of the PC. In confidence, they agreed that the major problems were the physicians’ lack of knowledge or skills in PC, and an inability to listen to them, of which, unfortunately, physicians remained unaware. Consequently, patients were not relieved of pain, no decisions about resuscitation were made, and no communication to patients and family members about the transition from curative to PC at end- of life was provided, which in turn led to patients dying alone. Therefore, they expected that the PCCT would give them support in their thinking about PC, especially in telling physicians how and when to decide about PC.

Initially, most team members were positive to the PCCT. However, when the PCCT started to ask questions about their routine work, some changed their positive attitude and questioned the PCCT’s understanding of the curative culture, their right to be on their ward, their medical competence and their right to get involved in “their patients” tests, treatment, and diagnosis. This became apparent during rounds, where the mood could quickly drop.

*There was an old man, around 90 years, and the physicians said that they would put in a tube and measure this and that. My spontaneous thought was- what does he want himself? I asked dr IMW 3, don’t you ever ask what the patients want. Then he stretched out, looked wide-eyed and said: No, we usually don’t do that. After the round I was treated like, you’re wrong out there! That’s also what the nurse told me afterwards. I felt pathetic.* Reflection among the PCCT.

#### Valuation of time and prioritising

In the acute wards, a curative performing culture was in focus, where working fast and effectively was the goal of care with the intention of doing a good work. Diagnosing and treatments were the first priorities and then planning for patients discharge. The value of fast work was obvious in the hectic ward environment, where team members hurried between different rooms and patients, indicating lack of time.

*Researcher: I wonder what you thought about the round today?*

*Primary nurse IMW 5 (F): What I was thinking about was that I had so many other things to do as well as a meeting for care planning... It takes too much time … at the same time, there are questions that should be discussed… but at the same time, you are torn since you want to listen, but you do not have time.* Interview with primary nurse after the round.

The PCCT noticed the lack of time and the following values problem among all primary team members, and felt overwhelmed, especially as regards patient participation, patient autonomy, and the lack of ethical reasoning and notice in patients’ and their families’ needs. Decisions were made without patients’ presence, and then delivered to them during rounds. This was something they were not used to, as every change made regarding care should be discussed with the patient and his/her family, so that everyone knew why. The PCCT claimed that communication was time saving in a longer perspective. The primary team members rational thinking was on doing as much as possible, “the maximum care”, regarding medical interventions rather than reflecting on the benefits for each patient, giving each deteriorating patient “a chance”. The PCCT was used to that such a decision required reflection and time so that the care was optimal for each patient, rather than maximal, but time was lacking in the acute culture.

In communications between PCCT and the primary team members, it was clear that when primary team members were working with severely ill and dying patients, they expected them to tell them if there were any problems, without actively asking patients themselves. They used their clinical view, i.e., if patients grimaced when being touched or moved, they might have some kind of distress. On rounds, the PCCT tried to convince them to use tools/scales to identify and continually measure symptoms, but they hesitant, as this was time-consuming.

*PCCT, nurse X (F): Have you tried to measure this patient’s symptom?*

*(It becomes silent. The question is unpleasant. Both the nurse and the assistant nurse wince. It becomes obvious that they have not asked the patient)*

*Primary nurse SW 4(F): No, but he does not have any symptoms… (The answer is hesitant and uncertain. She turns to the assistant nurse for confirmation.)*

*Primary assistant nurse SW 5(F): Nooo (hesitates)… he has never said anything about that.*

*(There is a silent agreement between the nurse and assistant nurse that this patient do not have any symptoms. The round continues.)* Observation from a round.

*After this round, the PCCT measures this particular patient’s symptom, and he estimates his symptoms to 8-10 on a VAS-scale, pain, nausea, anxiety and so on.* Following notice.

*The PCCT arrives to the ward. The head nurse meets them immediately.*

*Head nurse SW 22 (F) (troubled): The nurses are very distressed by “ESAS” (Edmonton symptom assessment scale) as they do not have time to use it. Their patients are very ill.*

*PCCT, nurse X (F): We do not use this scale for our sake, but for the patients’ sake. It is an aid and the nurses can use it whenever they want, when they talk to patients. It is not an obligation, but was on request by the nurses on the other ward.* Observation from a ward.

#### The content and creation of palliative care

The content of PC, and how to achieve good PC was interpreted quite different between the PCCT and primary team members, which made the different teams to collide. Primary team members defined PC as a short time in a patient’s life, a few hours before he/she dies. Then the patient is given pain-relief and eventually other drugs to relieve symptoms. Continually, the primary team members wanted to ensure the PCCT that they could not practice specialised PC, since they did not have time to do so. The PCCT were familiarised that PC should start earlier as the planning of the last care phase was incomplete; they had observed several cases when patient’s status deteriorated, death neared, and the patient became unconscious before any discussion about PC arose. No one spoke with the patient and his/her family, and knew nothing about their preferences regarding death and dying. This was not in line with the PCCTs’ view of good PC and made them frustrated.

*We usually don’t sit down with the patient and the family and talk about “where are we going”, talk about the future. Sometimes it’s like “oh, he’s dying” and then we give medications.* Interview with nurse SW 32.

The PCCT wanted planning and discussion with the whole team for the patients last days or weeks in life with the family present. But on rounds, there was seldom a consensus around the different primary team members, but rather divided views whether the patient was dying or not. On rounds, unpleasant decisions about PC were postponed or avoided, sometimes just an hour before the patient’s death, as the primary team did not have time for reflection around the patient’s whole situation and wanted to do as much as possible before “giving up”. Among some primary physicians, there was a wish to be positive in the meeting with the severely ill patients; they wanted to see the possibilities for each patient by remembering previous unbelievable recoveries. There was also a fear among primary team members of being criticised for making the wrong decision regarding PC, of not having done enough and examined a patient fully.

*PCCT consultant A (F): If you discover a malignancy, do you know what will happen then?*

*Primary consultant IMW2 (M=male): Not much, they cannot operate on her.*

*PCCT consultant A (F): Do you know what she wants herself?*

*Primary consultant IMW2 (M): No. Nevertheless, yesterday we started laxation, so we have to continue with the examinations. I’ve no one who stands behind me, you have your boss, I’ve no one.* Observation from a round.

*Primary consultant IMW4 (M): In this case, we have to guess that it is a possible tumour, that’s what the symptoms tell us. It’s not relevant with anything more (examinations), it’s just good nursing care. However, against all odds, you should never say never. I’ve seen people who have recovered the seemingly most impossible. But you should not hope for it, it looks very, very gloomy.. if nothing miraculous happen.* Observation from a round.

The PCCT was not used to provide blood infusions, artificial intravenous support and so on to the patient near death; instead, their experience were that it gave the dying patient more symptoms and decreased their quality of life. On the other hand, the primary team members believed that doing as much as possible was the same as doing the best for the patient near death to get patients better. “To not do anything” felt threatening.

*There was a dying woman on the round whom they decided to give blood. Although they had been visiting her, and seen her, they still prescribed this. She was in the terminal phase her breathing was irregular. Therefore, I asked if they realised that she was dying, and the goal of their treatment. The goal was “to make her a little more alert”. I talked about the dying process, the thinking around it, and her needs right now!* Diary, PCCT, nurse X (F).

*A woman with generalised cancer, over 80, who lost her appetite. The two nurses reported that they should make a cost registration. I asked why. Then it all started! They said of course, we have to make a registration, as we do not know how much she eats. That was a good thought to make things clear, but what will they do with the results? Then the physician became irritated: we cannot starve her to death! He became fiery. In addition, I commented: but that is what her disease will do with her anyway. However, he replied we could not starve her to death, can we?* Diary, PCCT, nurse X (F).

Instead of using the term “PC”, primary team members had their own definition of care, not as definitely as PC and easily changed if someone should complain that they did not do enough. This was intensely discussed during rounds.

*PCCT, consultant B (F): What you say now is very important; how you see, what PC is will influence your decisions.*

*Primary consultant IMW 10 (M): Well, that is the dilemma here.*

*PCCT, consultant B (F): This man cannot be healthy again, he is incurably ill, but you still have not reach the breaking point.*

*Primary consultant IMW 10 (M): No (firm).*

*PCCT, consultant B (F): Still you want to relieve his symptoms and improve quality of life.*

*Primary consultant IMW 10 (M): Yes, we use restricted, limited care, which is what it is (hesitates).*

*PCCT consultant B (F): Then it is obvious (annoyed).*

*Primary consultant IMW 10 (M): Many patients is not in the terminal stage and do not get PC.*

*PCCT, consultant B (F): But they ought to (determine).* Observation from a sitting round.

## Discussion

This study contributes detailed insight regarding cultural differences when team members in two different care cultures meet. The main finding of this study is that the PCCT and acute care have different knowledge and skills regarding PC and that there are resistance regarding PC in the acute wards when it comes to change routines. Consequently, this will contribute to the quality of patients’ end-of-life care.

The challenges for the intervention were many, including primary team members’ view of the skills regarding PC, their internal values as lack of time as well as the content of PC. This led to a collision between the PCCT and them, as PCCT noticed the lack of a holistic view on dying patients. The lack of time has also been reported in other studies [4, 5, 38–40]. This study shows that, even though the curative culture is intense and active, the primary team members have no time to reflect on each patient’s holistic wellbeing or lack thereof, which may lead to care that is not entirely secure for dying patients. This is also a question of priorities. Acute wards have dying patients, and according to Swedish law [41], care must be given with respect to the equal value of all human beings and for the dignity of the individual human being. Therefore, a PCCT is needed, since care should be adapted to the needs of all inpatients, not only those who will recover. Moreover, it is not possible to have specialised wards for every diagnosis or condition. Being medically active for a patient at the end of their life can diminish quality of life and hinder the opportunity to end his/her life with dignity, as well as leading to consequences for the family’s grieving. It may also result in unnecessary costs for examinations [19, 42]. Nevertheless, the time of death is challenging to predict as well as it is difficult to be secure when the dying process start. However, there are clear symptoms and signs on this; anorexia-cachexia, dysphagia, and delirium lessens oral intake [43] and most symptom scores increase during this phase such as dyspnoea, fatigue, drowsiness, xerostomia and hallucinations [44].

Interestingly, some primary team members had their own name for PC, so-called restricted or limited care, a kind of “PC light”, and restricted the term PC to care in the last hour of life. The latter has also been reported in another study [45] where physicians, even after training, continued to associate PC with the terminal or dying stage. This shows how difficult (but not impossible) it can be to meet another culture with another kind of thinking and routines.

In the current study, primary care nurses were more interested in PC than physicians were assuming it did not interfere with their own practice. A review study focusing on nurses in acute care hospitals showed that nurses felt a commitment to help dying patients to a good death, and to share the end-of-life experience with patients/families. The challenge was managing the different needs of curative and PC patients in a biomedical culture of care. Nurses’ ideals of good PC care were not recognized and supported by the organisation [39], which is important. However, nurses in the current study had limited control of which patients who were dying, and continued with the curative intention, for example regarding nutrition.

Primary team members in acute wards continually receive training in resuscitation, as this is a main and prioritised part of a curative culture. Still, they usually do not receive PC training, although many patients die in their wards. This mirrors the perspective and thinking of the culture. In the future, there will probably be a need for a PCCT to integrate PC as a natural part of care, and for at minimum annual PC training on acute wards. Health care should be secure and safe for all consumers, e.g., patients and family members. As long as acute care wards treat dying patients, they have a responsibility to provide the best care for them, too. This may also be a question of experience, as one study showed that nurses with postgraduate training perceived significantly fewer barriers towards end- of- life care than those inexperienced in caring for dying patients [46]. Another study showed that primary team members thought that PC can be perceived as a last resort, and do not want to believe themselves or suggest to their patients that future treatment is futile [21].

There are a number of methodological limitations to our study. Is it possible to undertake an ethnographic study of different cultures such as internal medicine wards and surgical wards? They may not have the same culture, even though there are studies that show that it is the organisational culture, and its attitudes, that colours the ward’s atmosphere [47, 48].

One can question Dewey’s ideas regarding knowledge increases in social processes, and changes without the appearance of chaos. This statement was not fully confirmed by this study regarding the chaos reasoning, since there were several collisions during the intervention. When different organisational cultures meet, this is almost inevitable, as seen in other studies [21, 38]. Thus, transferability of our findings to other health care systems or different organized institutions is possible as there are other studies despite of country origin that partly have found comparable results [1, 5, 49]. The strength of this study is the large volume of data collected and the different data that have provided rich material. The first author’s research area is PC, and the data analysis might been influenced by that. We used researcher triangulation and member checking which establish credibility and contribute to trustworthiness.

To conclude, this study shows the differences when two different care cultures meet and attempt to work together. Primary team members in acute care settings may provide good PC, but this study report that this still is a challenge. Cultural routines and thinking are challenged, but can be overcome.

## Data Availability

The data are not publicly available due to them containing information that could compromise research participant privacy/consent. If you have any questions or comments with regard to the availability of the data and materials, please contact the corresponding author (MF).
